# Physiological and transcriptomic analysis reveals the potential mechanism of *Morinda officinalis* How in response to freezing stress

**DOI:** 10.1186/s12870-023-04511-5

**Published:** 2023-10-23

**Authors:** Zhenhua Luo, Xiaoying Che, Panpan Han, Zien Chen, Zeyu Chen, Jinfang Chen, Sishi Xiang, Ping Ding

**Affiliations:** School of Pharmaceutical Sciences, Guangzhou University of Chinese Medicine, Guangzhou, 510006 China

**Keywords:** *Morinda officinalis* How, Freezing stress, Transcriptomics, Physiological analysis

## Abstract

**Background:**

*Morinda officinalis* How (MO) is a vine shrub distributed in tropical and subtropical regions, known as one of the “Four Southern Herbal Medicines” in China. The unclear responsive mechanism by which MO adapt to freezing stress limits progress in molecular breeding for MO freezing tolerance.

**Results:**

In this study, morphological, physiological and microstructure changes in MO exposed to -2℃ for 0 h, 3 h, 8 h and 24 h were comprehensively characterized. The results showed that freezing stress caused seedling dehydration, palisade cell and spongy mesophyll destruction. A significant increase in the content of proline, soluble protein and soluble sugars, as well as the activity of superoxide dismutase and peroxidase was observed. Subsequently, we analyzed the transcriptomic changes of MO leaves at different times under freezing treatment by RNA-seq. A total of 24,498 unigenes were annotated and 3252 unigenes were identified as differentially expressed genes (DEGs). Most of these DEGs were annotated in starch and sucrose metabolism, plant hormone signal transduction and MAPK signaling pathways. Family Enrichment analysis showed that the glucosyl/glucuronosyl transferases, oxidoreductase, chlorophyll a/b binding protein and calcium binding protein families were significantly enriched. We also characterized 7 types of transcription factors responding to freezing stress, among which the most abundant family was the MYBs, followed by the AP2/ERFs and NACs. Furthermore, 10 DEGs were selected for qRT-PCR analysis, which validated the reliability and accuracy of RNA-seq data.

**Conclusions:**

Our results provide an overall view of the dynamic changes in physiology and insight into the molecular regulation mechanisms of MO in response to freezing stress. This study will lay a foundation for freezing tolerance molecular breeding and improving the quality of MO.

**Supplementary Information:**

The online version contains supplementary material available at 10.1186/s12870-023-04511-5.

## Introduction

*Morinda officinalis* How (MO) is a perennial vine shrub and belongs to Rubiaceae family. The roots of MO have been often used as a tonic or traditional Chinese medicine attributed to its unique effects of tonifying kidney-yang, strengthening muscles and bones, and eliminating wind dampness [1]. Researches showed that MO contained many bioactive components, such as oligosaccharides, polysaccharides, iridoids and anthraquinones [2–5]. Among them, nystose of the oligosaccharide component had attracted extensive attention owing to its high content, anti-depression and anti-osteoporosis effects [6–8].

MO prefers a warm and humid climate, mainly distributed in tropical and subtropical regions of China such as Guangdong, Guangxi, Hainan and Fujian provinces. Deqing county in Guangdong is one of the genuine producing areas of MO, where is surrounded by hills, with abundant rainfall, sunshine and mild climate. In recent years, the demand for MO has increased dramatically and the wild resources have been on the verge of extinction. Most of MO had been propagated by cuttings presently. We have previously conducted a large number of investigations on MO germplasm resources, and found that there is rich genetic diversity in MO population [9]. According to the difference of leaf shape and leaf size, it is mainly divided into large-leaf type, middle-leaf type and small-leaf type. We have learned in practice through prolonged observation that the small-leaf type has the characteristics of high yield, strong disease-resistance and easy-planting, while the large-leaf type has the characteristics of cold-resistance and drought-resistance, both of which are widely planted in actual production.

As a result of long-term asexual reproduction, MO may be susceptible to diseases and virus, leading to the degradation of MO germplasm and quality [10–12]. In order to improve the situation of succession cropping obstacle, it is of great significance to try to migrate the MO cultivation area, which just like *Panax notoginseng* [13] and *Rehmanniag lutinosa* [14]. Interestingly, we found that when MO was transplanted to Wengyuan county, it still maintained its original growth characteristics, had great adaptability and a higher yield, indicating that the northward introduction was initially successful. The roots of MO produced by Wengyuan had numerous branches, short and thick, with a light purple cross-section [15]. Moreover, the nystose content in MO samples of different growth years generally increased. This may be benefited from the special geological conditions and climate of Wengyuan county, which is located in the north of Guangdong province, with fertile soil, a relatively lower annual average temperature and the minimum of -2℃ in winter. The plants usually adapt to freezing environment by accumulating sugars or changing their cell components [16–19]. However, due to the special growth characteristics of MO, unusual abrupt temperature changes in winter and later spring frost events could seriously attenuate MO growth, development and yield. Therefore, it is an urgent need to clarify MO freezing response mechanisms.

Freezing stress changes not only the phenotype of plants, such as slow growth, leaves dehydrated and atrophied, but also various intracellular metabolisms, such as slower membrane fluidity, increased membrane permeability and the occurrence of lipid peroxidation reaction [20, 21]. In the long-term evolutionary process, plants have formed a series of defensive mechanisms to adapt and resist to low temperature [22]. When plants are exposed to a nonlethal low temperature, cold signals are perceived by hypothetical sensors, including cell membranes, calcium (Ca^2+^) channels and G-protein regulator (COLD1) [23–25]. The concentration of intracellular Ca^2+^ increases briefly, which stimulates the signaling process. The promoter of the cold regulation (COR) gene contains a CRT/DRE cis element that can bind to DREBs. DREBs include *DREB1/CBF* and *DREB2*, which would be induced by low temperature and dehydration, respectively [26]. Also, the CBFs could be activated by other transcription factors of upstream elements, such as the *ICE* protein [27]. In addition, plant hormone plays a fundamental role in regulating plant responses to low temperature. For example, the increase content of abscisic acid (ABA) would close the stomata, reduce water loss, activate downstream signals and finally enhance the tolerance to dehydration and low temperature. Ca^2+^ signals at the forefront of signaling process can also be transmitted by ABA signals. ABA may regulate the expression of COR gene through pathways that affect CBFs or unrelated to CBFs [28]. Through the integration and regulation of low-temperature signal transduction networks, plants ultimately improve their cold-resistance ability by changing their physiology and internal structural characteristics.

Higher lipid content and unsaturation are positive factors to improve cold tolerance by maintaining the integrity and fluidity of plasma membrane [29]. The thickness of leaves, palisade tissue and sponge tissue usually vary with the natural environment and physiological conditions of plants, and the changes in internal structural of leaves are often used as one of the important criteria for freezing tolerance. Proteins involved in the stability and repair of photosynthesis play an important role in maintaining the normal structure and function of chloroplasts [30–32]. Furthermore, low temperature can induce the accumulation of cell osmore-gulation substances, such as proline, soluble sugar and soluble protein, to prevent the decrease of intracellular water potential. In addition, antioxidant systems such as superoxide dismutase (SOD), peroxidase (POD) and catalase (CAT) play a central role in removing excess ROS [33].

Presently, little is known about MO freezing response mechanism. In this study, we comprehensively characterized the morphological, physiological and microstructural changes to freezing treatment in MO. The large-leaf type of MO was selected for analysis since it showed a higher freezing tolerance than the small-leaf type in our previous preliminary study. We also utilized RNA-seq to evaluate changes in the expression of DEGs at different time points, and identified several genes that may strongly impact the freezing resistance process. It is the first report exploring the MO freezing response molecular mechanism. Our results can provide valuable resources for practitioners performing MO freezing tolerance breeding, laying the foundation for expanding MO cultivation area and improving the quality and yield of MO.

## Materials and methods

### Plant material

The four-year-old MO plants were collected from Deqing county, Guangdong province of China. The samples were identified by Prof. Ping Ding (Guangzhou University of Chinese Medicine, Guangdong, China). In this study, all seedlings were produced by the tissue culture methods, which is a stable genetic system established in the previous study [34]. The original vines of MO were cut from the stem base, and new vines grew after one month in a clean greenhouse. Young stem segments of MO were used as explants and inoculated into Murashige and Skoog (MS) medium (3% sucrose, pH6.0, 0.4% agar, 1.0 mg/L 6-benzyladenine and 0.5 mg/L indole butyric acid) after disinfection. When the tissue culture seedlings grew for 60 days, they were transferred to pots filled with a 1:1 mixture of perlite and peat soil. The culture temperature was set at 22 °C, photoperiod 12 h/12 h (light/dark), light intensity of 1200 lx, and humidity of 75%.

### Freezing treatment, phenotypic observation and physiological indexes evaluation

The MO seedlings were transferred to a variable-frequency refrigerator for -2℃ freezing treatment when they continue to grow for 30 days under normal conditions after been transplanted. 15 seedlings were treated and repeated three times. The apically third leaves were collected at 0 h, 3 h, 8 h, and 24 h, with three biological replicates at each time point. All materials were quickly frozen with liquid nitrogen and stored in -80℃ refrigerator for physiological index measurement.

Fresh leaves were collected and the relative electrolyte leakage (REL) was determined by a digital conductometer (Lichen, CT-20). The content of soluble sugar (Solarbio, Beijing, China), soluble protein (Geruisi, Suzhou, China), malondialdehyde (MDA, Jiancheng, Nanjing, China), proline (Real-Times, Beijing, China) and chlorophyll (Leagene, Beijing, China), the activities of superoxide dismutase (SOD, Jiancheng, Nanjing, China) and peroxidase (POD, Jiancheng, Nanjing, China) were measured using the kits. All parameters were measured by an enzyme-labeled instrument (A51119700DPC) with three technical replications.

### Microstructure observation

Samples were taken near the main veins and cut into 3 × 3 mm pieces. After completely immersed in FAA fixative solution (5% formalin, 5% glacial acetic acid and 90% ethanol) for 24 h, the series alcohol dehydrated, xylene transparent, paraffin embedded, sectioned, safranin fast green stained, placed under an optical microscope for observation and photograph. The thickness of leaf, palisade tissue and sponge tissue were measured (ImageJ V1.8.0.112). Then the organizational structure closely degrees (CRT, thickness ratio of palisade cell to leaf), organizational structure loose degrees (SR, thickness ratio of sponge tissue to leaf), and P/I value (thickness ratio of palisade cell to sponge tissue) were calculated.

### Total RNA extraction, library construction and transcriptome sequencing

Total RNA was extracted using the TransZol Up Plus RNA Kit (Transgen, Beijing, China) according to the manufacturer’s instructions. The concentration and purity of the RNA were assessed with the NanoDrop 2000 (Thermo Scientific, USA), and the RNA integrity was measured by the Agilent 4200 Bioanalyzer. Qualified RNAs were taken for cDNA library construction using the NEBNext® Ultra™ RNA Library Prep Kit for Illumina® (NEB, USA). Then sequencing was carried out using the Illumina Novaseq 6000 platform (Illumina, USA) with a paired-end reads length of 150 bp at Science Corporation of Gene (Guangzhou, China). The amount of sequencing data per sample was not less than 6 Gb.

### Transcriptome assembly, annotation and differential expression analysis of genes

The sequencing results were transformed into raw sequences after base identification by CASAVA. The raw reads were first processed through in-house Perl scripts, and high-quality clean reads were obtained after filtering out the reads containing connectors and low-quality reads. High-throughput sequencing data were assembled into transcript using the Trinity strategy. Unigene annotation was based on Nr (https://www.ncbi.nlm.nih.gov/), Uniprot (https://www.uniprot.org/), KEGG (https://www.kegg.jp/.) and KOG/COG (https://www.ncbi.nlm.nih.gov/COG/) databases [35–38]. The clean reads of each sample were compared to the assembled non-redundant transcripts using hisat2 v2.1.0 [39]. The TPM and FPKM values of each transcript were calculated by stringtie v1.3.3b based on the comparison results, with |log_2_FC|≥ 2 and *P* < 0.05 as the screening criteria for significantly differentially expressed genes [40]. They were subjected to GO, KOG and KEGG enrichment analysis, and entries with *P* < 0.05 were considered significantly enriched.

### qRT-PCR analysis for differentially expressed genes

To verify the reliability of transcriptome results, 10 significant DEGs associated with freezing response were screened for qRT-PCR analysis in this study. The total RNA of the samples was reverse transcribed using the TransScript Uni All-in-One First-Stand cDNA Synthesis SuperMix for qPCR kit (Transgen, Beijing, China). According to the manufacturer’s instructions, the qRT-PCR was carried out with a qtower3 real-time PCR instrument (Jena, Germany) using the Perfect Start Green qPCR Super Mix kit (Transgen, Beijing, China). Transcript levels were normalized against the average expression of the *CYP* gene. All primer sequences were listed in Table S1. Each reaction contained 10 μl of 2 × Perfectstar green qPCR Supermix, 0.4 μl of the forward primers (10 μM), 0.4 μl of reverse primers (10 μM), 2 μl of the cDNA and finally made up to 20 μl with nuclease-free water. Three biological and technical replicates were performed for each sample. The qRT-PCR reaction system was as follows: initial denaturation at 95 °C for 30 s, 40 cycles of denaturation at 95 °C for 5 s, and annealing at 60 °C for 34 s. The relative expression levels of target genes were calculated using the 2^−ΔΔCt^ method.

### Statistical analysis

Statistical analysis was performed using analysis of variance (ANOVA) followed by Duncan’s multiple range test (SPSS 25.0, SPSS Inc., IL, USA). ^*^P < 0.05, ^**^P < 0.01 and ^***^P < 0.001 represent significant differences at the 0.05, 0.01 and 0.001 levels, respectively.

## Results

### Phenotypic changes to freezing treatment

**Fig. 1:**
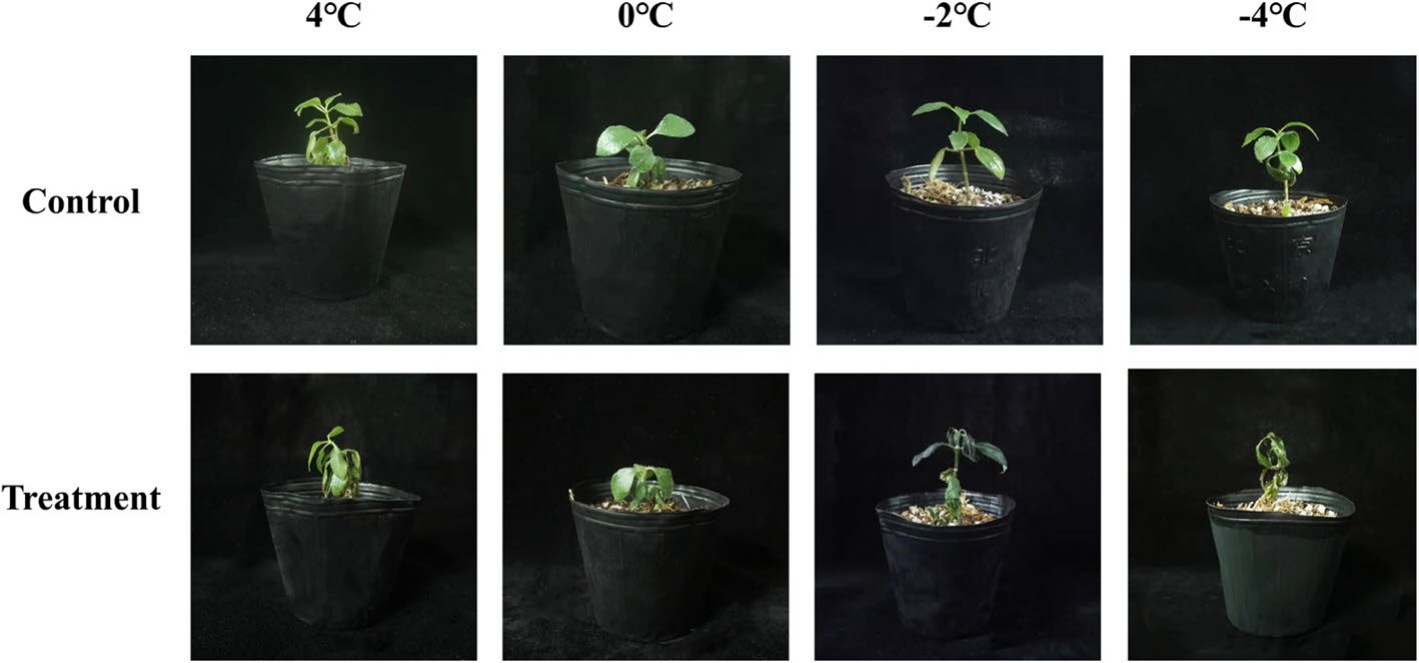
The morphological characterization of MO seedlings under different freezing treatments (4, 0, -2, -4℃)

**Fig. 2:**
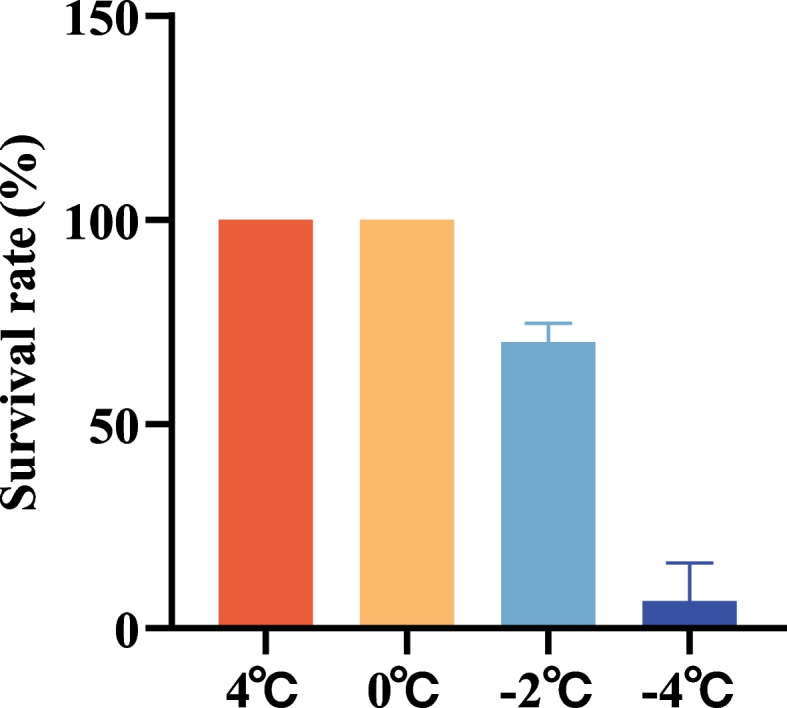
The survival rate of MO seedlings under different freezing treatments

We explored the optimal freezing conditions of MO seedlings (Fig. 1). The leaves of MO seedlings were slightly wilted when frozen at 4 °C and 0 °C for 24 h but recovered when transferred to room temperature for 48 h. The damage degree to seedlings deepened with the decrease in temperature. After 24 h of freezing stress at -2 °C, the leaves and petioles of MO were severely dehydrated and wilted. Some of them could not recover when placed at room temperature for 48 h, and the average survival rate of MO was 70.0% (Fig. 2). Freezing at -4℃ for 24 h, MO seedlings were almost dehydrated and did not recover. Therefore -2 °C was selected as the treatment condition for the subsequent experiment.

**Fig. 3:**
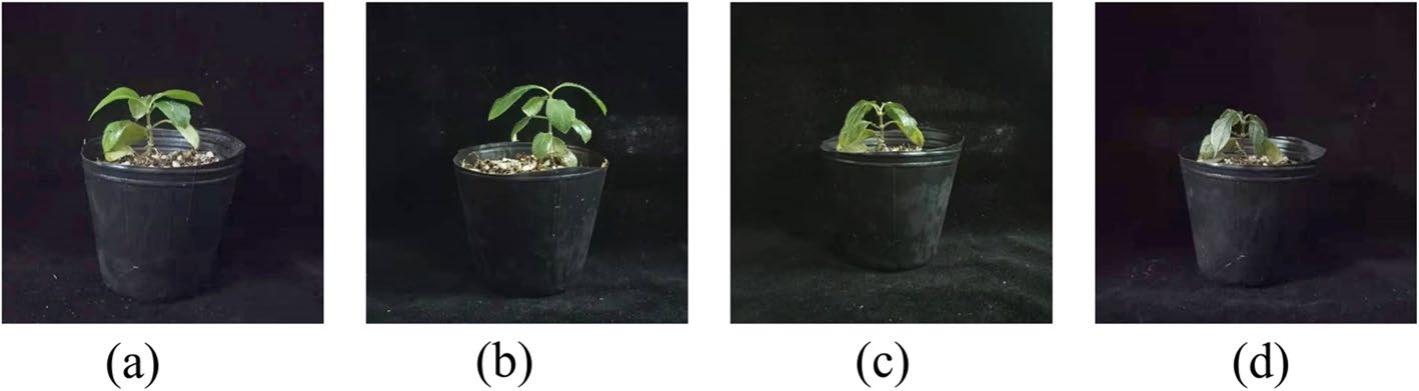
The morphological changes of MO seedlings at -2℃ treating for **a** 0 h, **b** 3 h, **c** 8 h and **d** 24 h

We also found differences in the morphological changes of MO when exposed to -2℃ for 0 h, 3 h, 8 h and 24 h. The leaves of MO seedlings showed mild freezing injury symptoms at the early stage (3 h). After 8 h freezing treatment, the petioles of MO seedlings began to dehydrate and wilt. At 24 h, they had apparent freezing injury symptoms, even lodging occurred in some plants (Fig. 3).

### Microstructural changes to freezing treatment

**Fig. 4:**
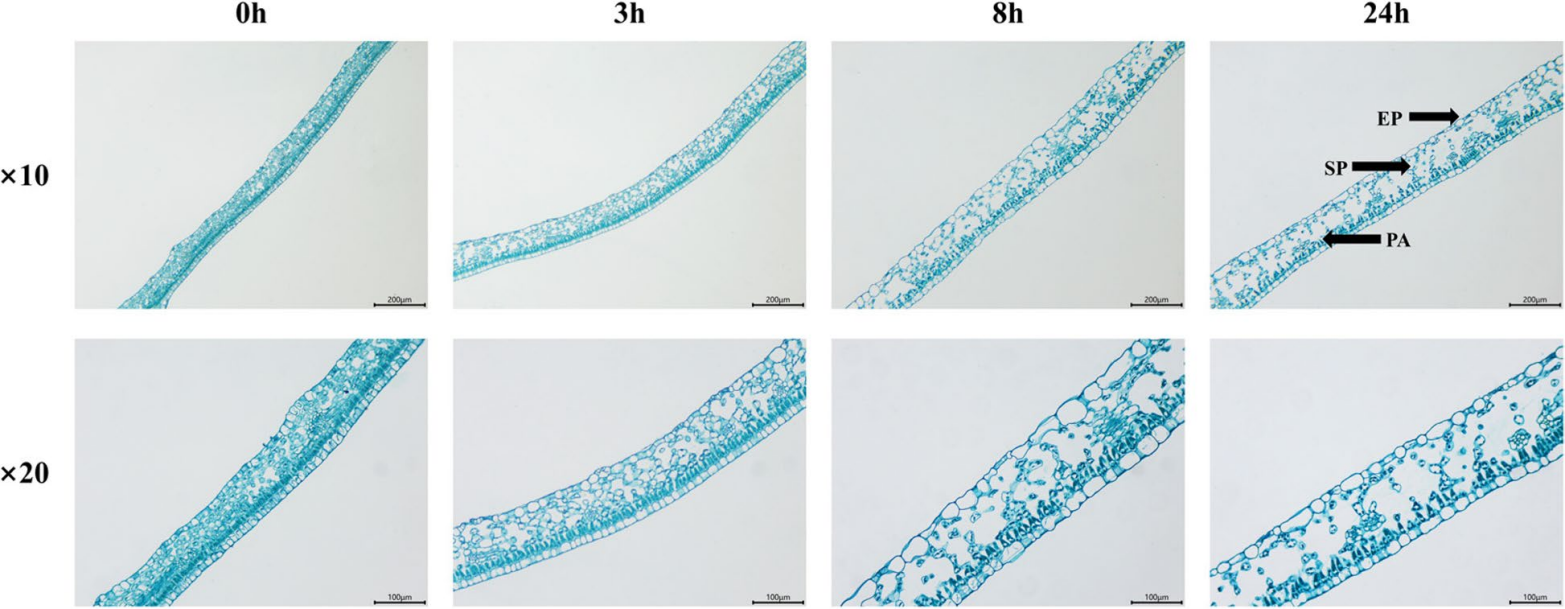
The microstructural changes of MO leaves at -2℃ for different freezing times (0, 3, 8, 24 h). EP: epidermal cells, SP: spongy tissues, PA: palisade cell. × 10 and × 20 represent magnifications are 10 and 20

**Fig. 5:**
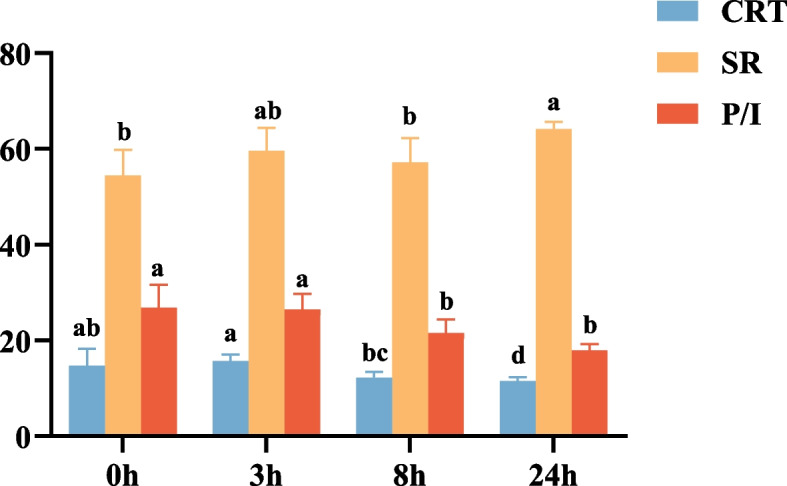
The value of CRT, SR and P/I changes of MO leaves at -2℃ for different freezing times (0, 3, 8, 24 h). CRT: organizational structure closely degrees, SR: organizational structure loose degrees, P/I: the thickness ratio of palisade tissue to sponge tissue

We further observed the microstructural changes of MO leaves under freezing treatment. As shown in Fig. 4, the palisade and sponge cells of MO were neatly arranged at 0 h. There was little difference between the treatment group (3 h) and the control group. Stressed for 8 h, the spongy tissues were sparse and the palisade cell were reduced. After continuously stressing for 24 h, the spongy cells relatively crumpled into clusters and appeared cavities. The thickness of leaves, palisade tissue and sponge tissue of MO for different freezing times was measured (Fig. 5 and Table S2). Results showed that organizational structure closely degrees (CRT) and the thickness ratio of palisade tissue to sponge tissue (P/I) decreased, while the loose degrees (SR) increased.

### Physiological changes to freezing treatment

**Fig. 6:**
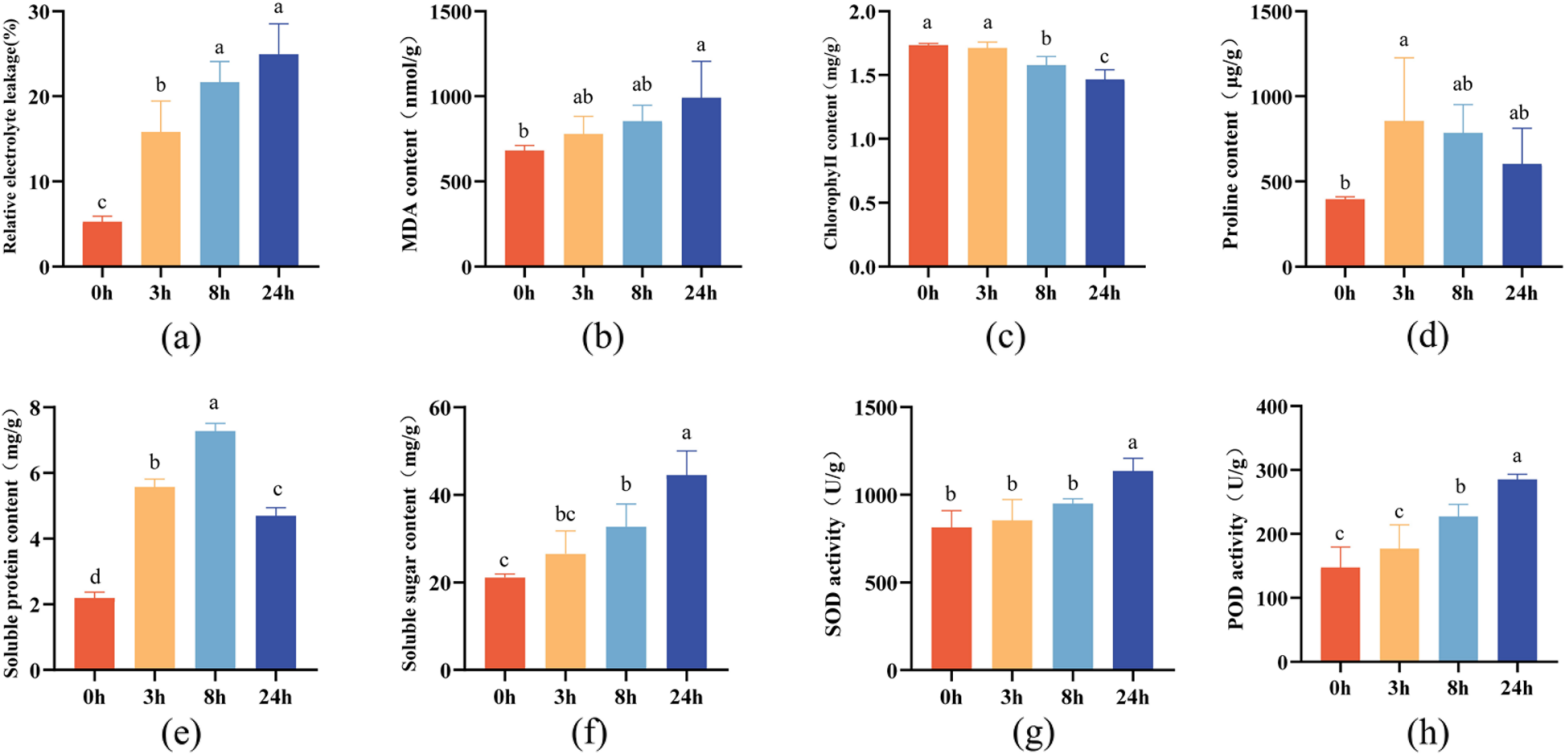
The physiological changes of MO seedlings at -2℃ for different freezing times. **a** Relative electrolyte leakage; **b** MDA content; **c** chlorophyll content; **d** proline content; **e** soluble protein content; **f** soluble sugar content; **g** SOD activity; **h** POD activity. The data represents mean ± SD (*n* = 3), and the different letters above the bar represent the values were significant difference with different freezing times (*P* < 0.05, Duncan’s test). U: Each milligram of tissue protein is defined as an SOD activity unit when the inhibition rate reaches 50% in 1 ml of reaction solution

The REL (Fig. 6a) and MDA content (Fig. 6b) of MO seedlings increased with the prolongation of freezing time, while the chlorophyll content decreased gradually (Fig. 6c), indicating that the continuous freezing caused irreversible damage to the biofilm. Freezing stimulation also promoted the accumulation of osmoregulation substances in plants to adapt to adversity. The content of proline and soluble protein increased with the extension of freezing time and decreased after 8 h (Fig. 6d and e). The soluble sugar content had no significant changes at the early freezing stage, yet sharply increased to 44.53 mg/g at 24 h (Fig. 6f). The antioxidant system also involved in MO freezing response process. The activity of SOD and POD in the treatment group was higher than in the control group (Fig. 6g and h).

### Transcriptome sequencing and assembly

To investigate the molecular mechanism related to freezing resistance of MO, we used RNA-seq approach to profile the leaves of large leaf species at 0 h, 3 h, 8 h, and 24 h exposure to -2 °C. In this study, 12 library reads were obtained. After filtering, clean reads varied from 19 to 27 million (Table S4). The content of GC was between 43.43% to 45.84%. Q20 value ranged from 96.87% to 97.31% and Q30 value range from 92.17% to 93.07%, indicating that the sequencing data were reliable.

The Trinity assembly strategy was used to splice the clean reads to obtain the reference sequences for subsequent analysis (Fig. S1). A total of 60,507 unigenes were obtained, with an average length of 1236 bp, N50 length of 2914 bp and N90 length of 423 bp. More sequences lengths in the range of 1 ~ 500 bp, indicating that the transcriptome data assembly was high quality and met the requirements for in-depth analysis.

### Functional annotation and analysis of genes

A total of 60,507 unigenes corresponding functional annotation information were obtained (Fig. S2), of which 24,425 (40.37%) were annotated in the UniProt database, accounting for the largest proportion. Following by 22,249 (36.77%) unigenes were obtained in the Nr database, 17,222 (28.46%) unigenes were annotated in the GO database, 13,101 (21.65%) unigenes were annotated to the KOG database, and 5661 (9.36%) unigenes were annotated to the KEGG database. There were 23,904 (39.51%) unigenes that had an annotation in at least one database. In addition, due to the unclear genetic background of MO, most unigenes (59.51%) had not been annotated.

According to the species distribution statistics of the most similar genes aligned in the Nr database (Fig. S3), *Coffea arabica* accounted for 7874 (47.22%), with the highest similarity. Followed by *Coffea eugenioides* accounting for 4100 (24.59%) and *Coffea canephora* accounting for 2548 (15.28%).

**Fig. 7:**
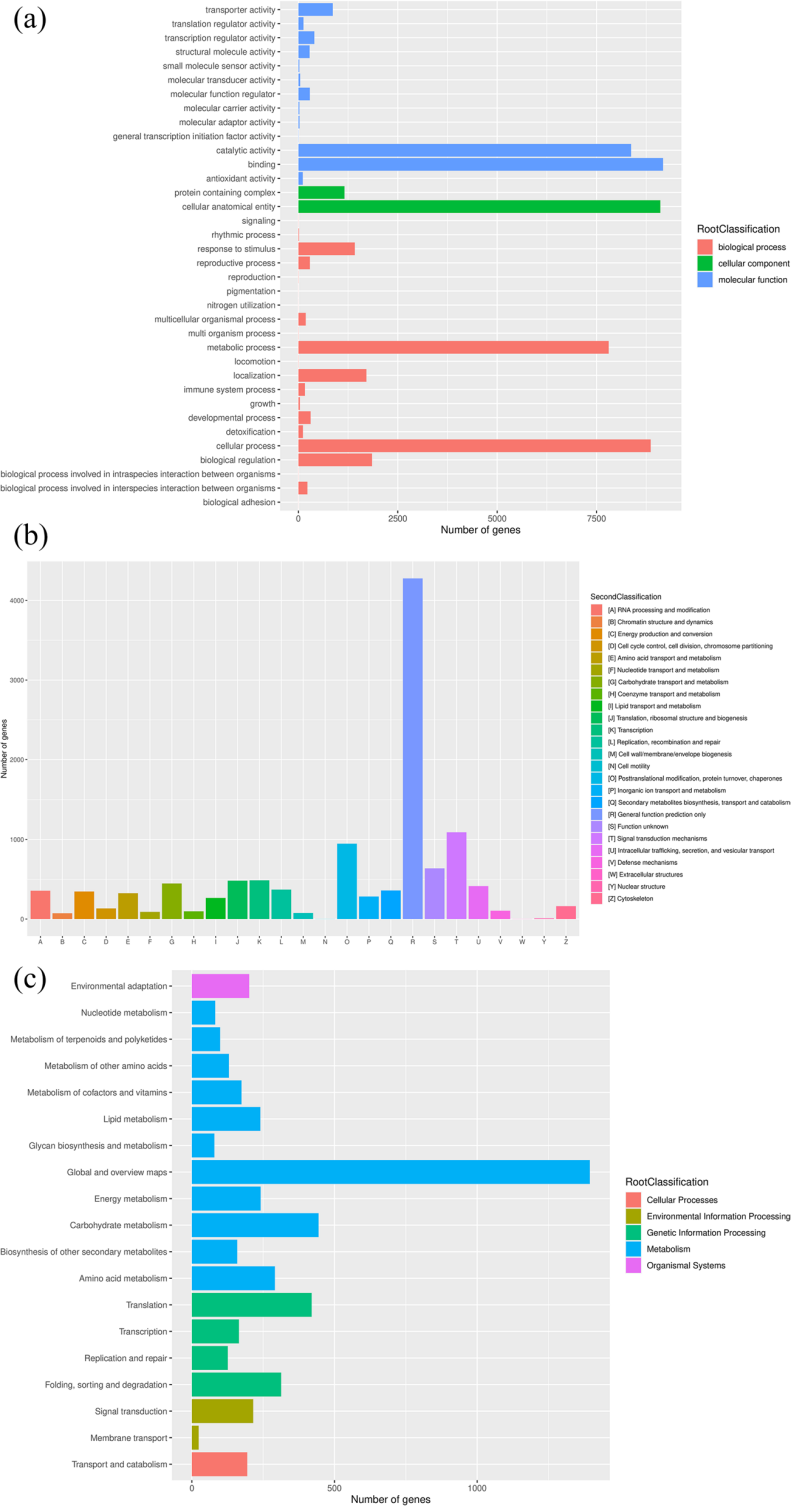
Functional annotation and analysis of unigenes. **a** The histogram of GO enrichment analysis; **b** The histogram of KOG enrichment analysis; **c** The histogram of KEGG enrichment analysis

In this study, the GO functional annotation results showed that 52,994 unigenes were classified into three major categories of biological processes, cellular components and molecular functions, and further divided into 36 subcategories (Fig. 7a). Among them, the unigenes involved in biological processes and molecular functions were the main components. In the biological processes, unigenes annotated as cellular processes had the largest percentage (8863), followed by metabolic process and biological regulation, which annotated 7895 and 1851 unigenes, respectively. The highest proportion of unigenes participated in cellular anatomical entity with 9105 unigenes in the cellular components category. In the molecular function category, unigenes accounting for the highest proportions were binding and catalytic activity, with 9174 and 8369 unigenes, respectively.

The KOG terms were assigned to 11,831 unigenes, of which functional annotation were divided into 25 categories (Fig. 7b). Most of them were distributed to cell processes and signals, including signal transduction mechanisms and posttranslational modification, with 1091 and 947 unigenes, respectively. In the information storage and processing classification, 487 unigenes were enriched in transcription. Importantly, there were 447 and 359 unigenes annotated to carbohydrate transport and metabolism, secondary metabolite synthesis, transport and metabolism in the metabolism category.

Blastp was used to compare all assembled unigenes sequences with the KEGG protein database to determine the most important biochemical metabolic pathway and signal transduction pathway involved in the genes (Fig. 7c). KEGG annotated 4992 unigenes mapped to 138 biological signaling pathways, mainly including metabolism, genetic information processing and environmental information processing. Among them, 444 unigenes were enriched in carbohydrate metabolism pathway, 291 unigenes were annotated to amino acid metabolism pathway and 240 unigenes were annotated to the lipid metabolism pathway.

### Screening differentially expressed genes

**Fig. 8:**
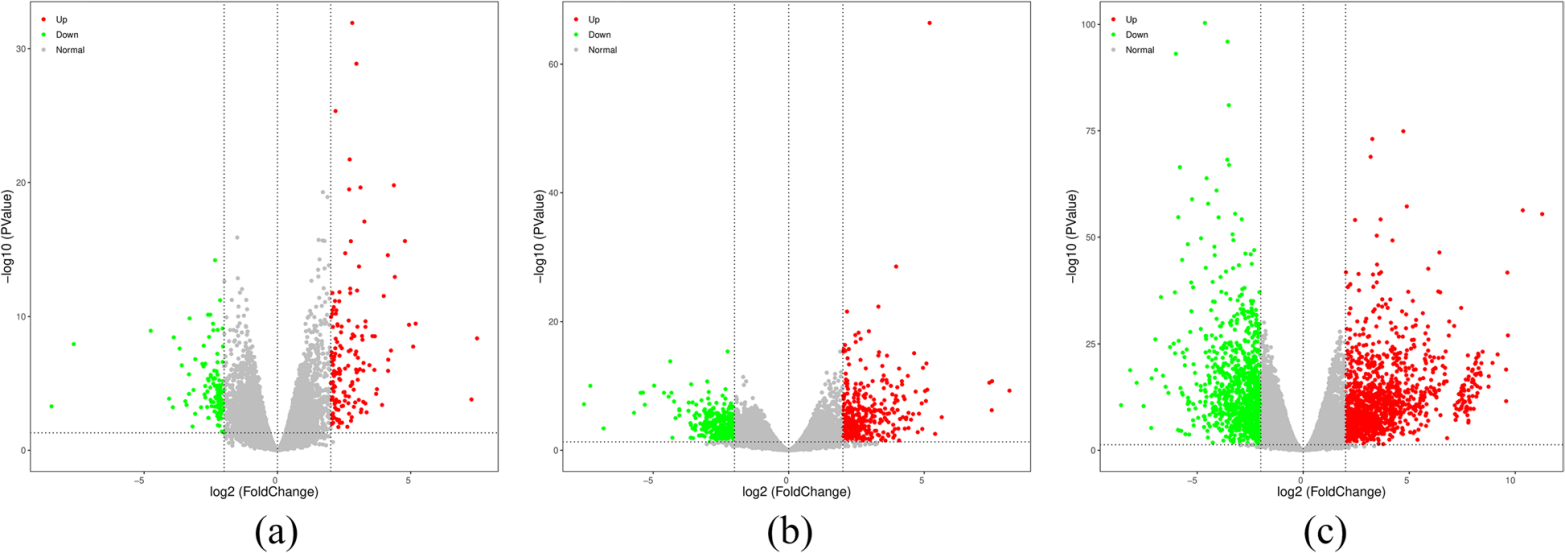
Number of DEGs in different freezing times. **a** 3 h_vs_0 h; **b** 8 h_vs_0 h; **c** 24 h_vs_0 h

To explore which resistance-responded genes related to improve the freezing stress, we constructed a comparative analysis of differentially expressed genes (DEGs) between the freezing-treated and control groups (Fig. 8). There were 257 (151 upregulated genes and 106 downregulated genes), 729 (357 upregulated genes and 372 downregulated genes) and 2266 (1265 upregulated genes and 1001 downregulated genes) DEGs were identified at 3 h, 8 h and 24 h, respectively, suggesting that the gene expression was less changed in the initial response stage of chilling stress.

### Functional analysis of differentially expressed genes

#### Family enrichment analysis of differentially expressed genes

**Fig. 9:**
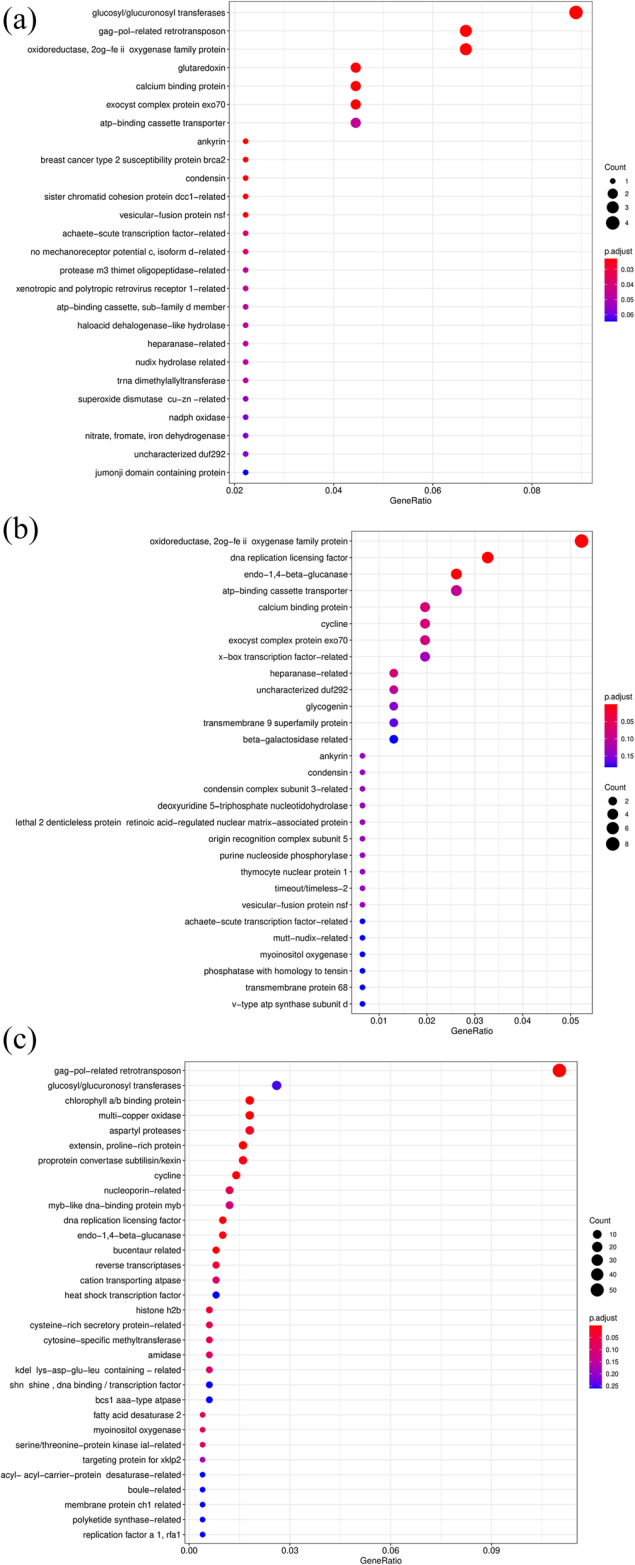
Family enrichment analysis of DEGs in different freezing times. **a** 3 h_vs_0 h; **b** 8 h_vs_0 h; **c** 24 h_vs_0 h

The results of family enrichment analysis showed that there were 26, 29 and 32 DEGs enriched in 3 h_vs_0 h, 8 h_vs_0 h and 24 h_vs_0 h group, respectively. Compared with the control group (0 h), the DEGs were significantly enriched in the “glucosyl/ glucuronosyl transferases”, “oxidoreductase” and “calcium binding protein” family after 3 h freezing (Fig. 9a). After 8 h stressing, the DEGs were enriched in “oxidoreductase”, “endo-1,4-beta-glucanase”, “calcium binding protein” and “glucosyl/glucuronosyl transferases” family (*P* < 0.1) (Fig. 9b). After 24 h continuous freezing, the DEGs were significantly enriched in the “glucosyl/glucuronosyl transferases”, “proline-rich protein” and “chlorophyll a/b binding protein” family (Fig. 9c). To sum up, the DEGs of MO were co-expressed in the “glucosyl/ glucuronosyl transferases” family under the three freezing stress stages.

#### KEGG enrichment analysis of differential genes

**Fig. 10:**
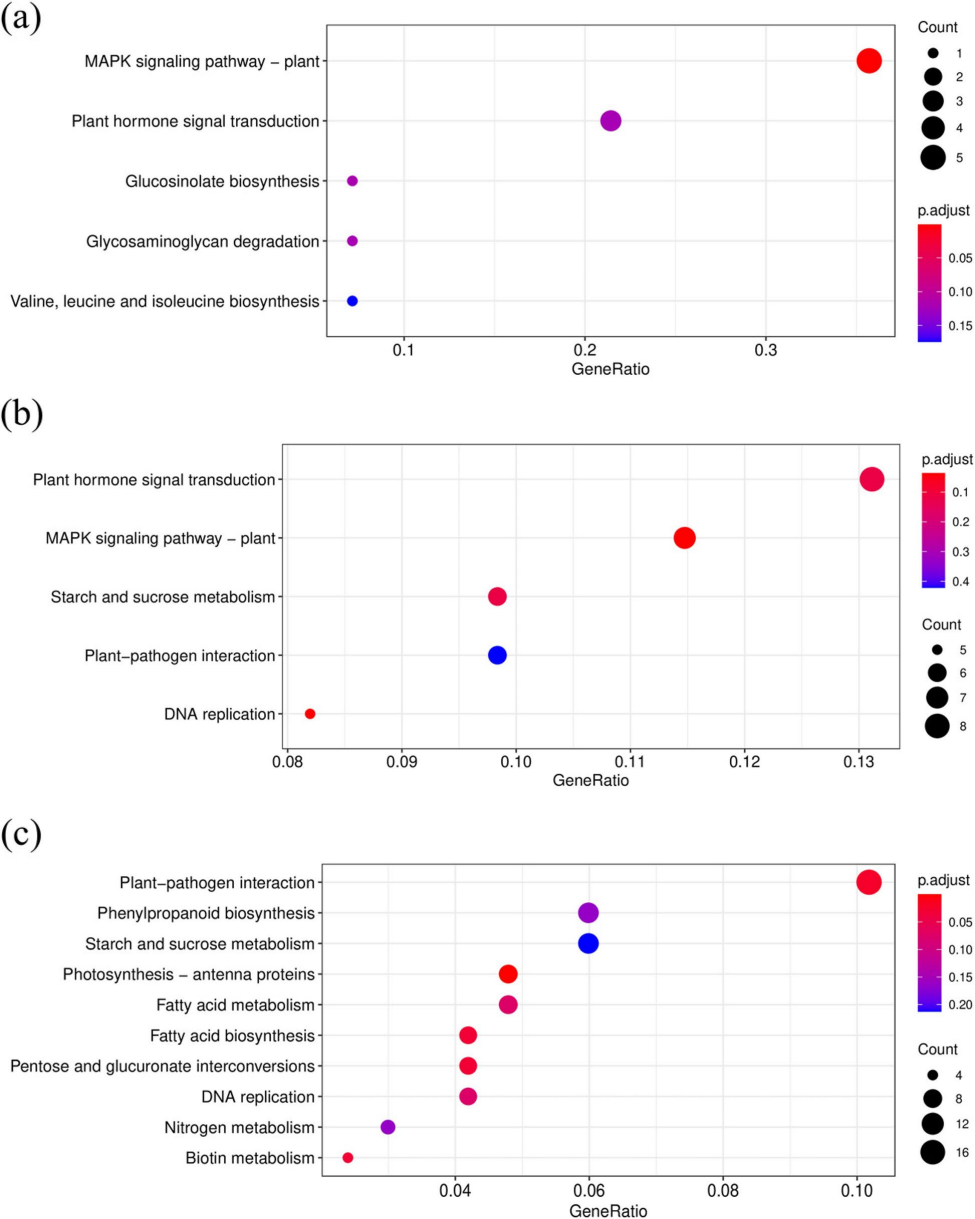
KEGG enrichment analysis of DEGs in different freezing times. **a** 3 h_vs_0 h; **b** 8 h_vs_0 h; **c** 24 h_vs_0 h

KEGG annotation showed that the DEGs were significantly enriched in “MAPK signaling pathway”, “plant hormone signal transduction” and “glucosinolate biosynthesis” at 3 h (Fig. 10a). At 8 h, the DEGs were significantly enriched in “plant hormone signal transduction”, “MAPK signaling pathway” and “starch and sucrose metabolism” (Fig. 10b). At 24 h, the DEGs were significantly enriched in the “plant-pathogen interaction”, “starch and sucrose metabolism” and “phenylpropanoid biosynthesis” (Fig. 10c).

**Fig. 11:**
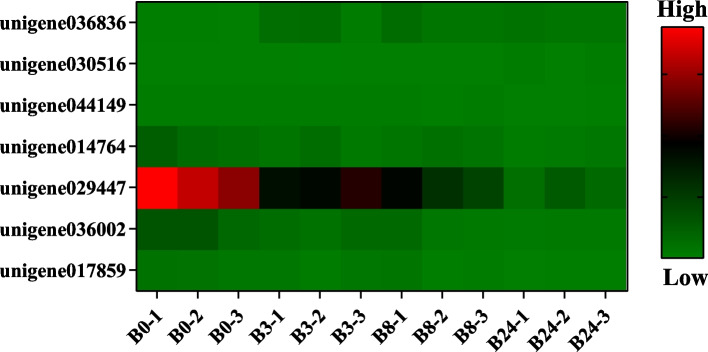
Heat map of DEGs related to plant hormone signal transduction pathway

**Fig. 12:**
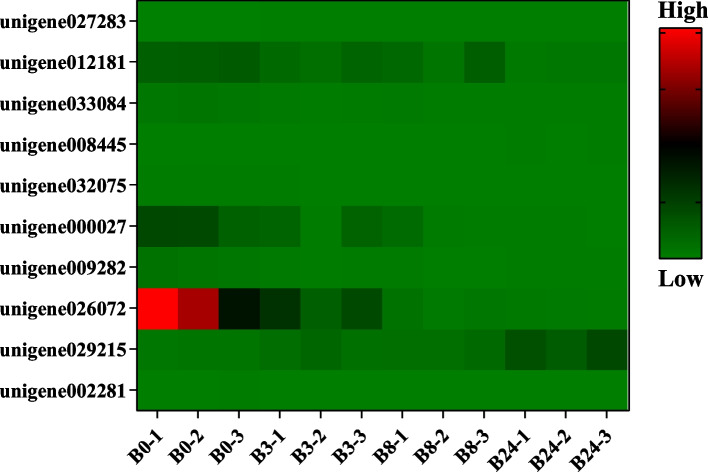
Heat map of DEGs related to starch and sucrose metabolism pathway

In the plant hormone signal transduction pathway, there were 7 DEGs whose expression levels varied significantly under freezing stress. These genes are mainly involved in the abscisic acid and auxin signaling process (Fig. 11). In addition, there were 10 DEGs enriched in the starch and sucrose metabolism pathway, which were mainly involved in starch degradation and trehalose synthesis (Fig. 12).

#### Differential expression analysis of transcription factors

**Fig. 13:**
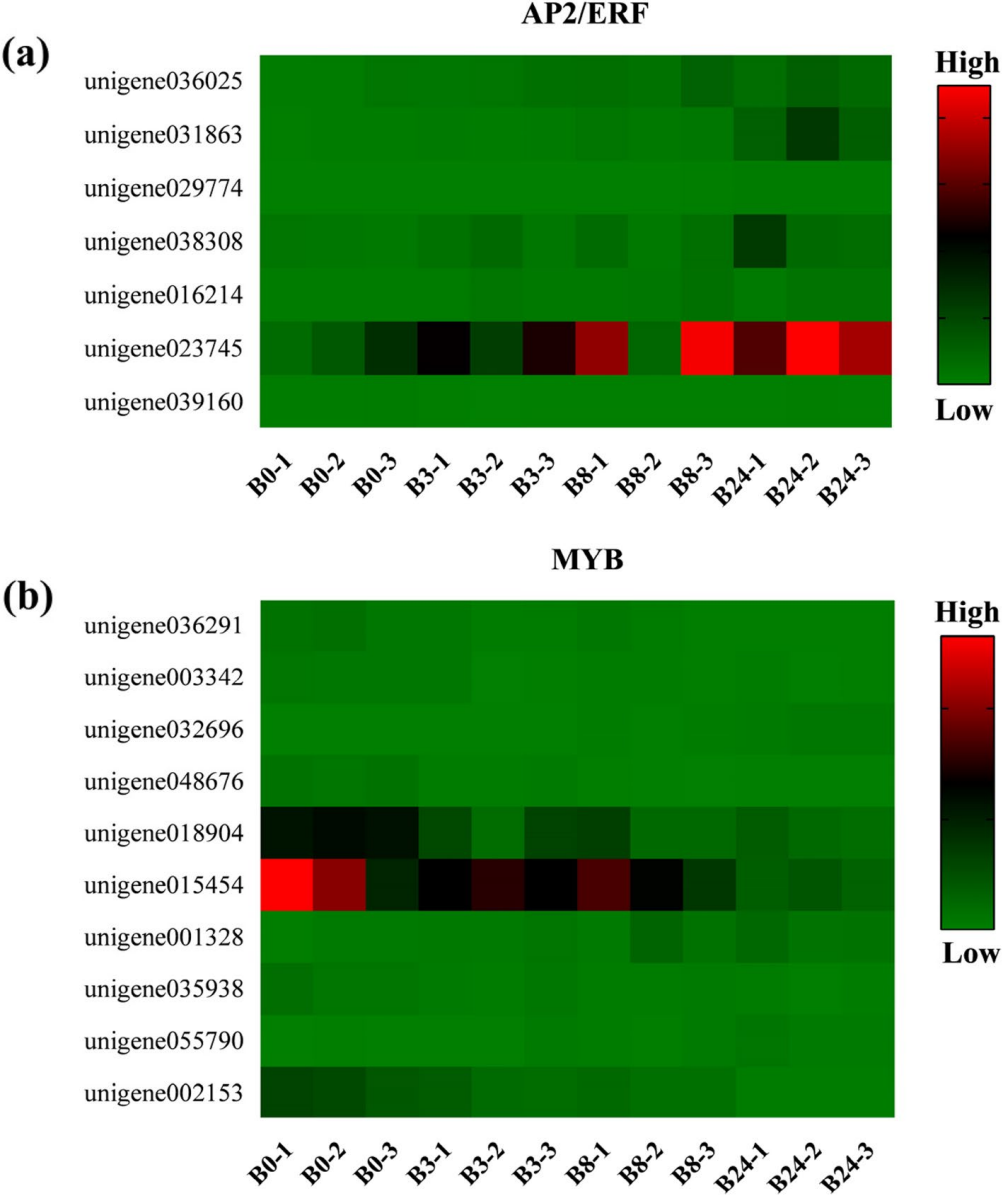
Heat map constructed based on FPKM expression values of **a** AP2/ERFs and **b** MYBs

There were 22 DEGs enriched in 7 main transcription factor types (Fig. 13 and Table 1), including 10 MYBs, 7 AP2/ERFs (RAVs, ERFs and DREBs), 1 NAC, 1 WRKY, 1 ZFP, 1 bHLH and 1 CAMTA. Among these differentially expressed transcription factors, the number of upregulated expression (13 genes) was greater than that of downregulated expression (9 genes). Among them, the expression levels of unigene03186, unigene039744, unigene032696, and unigene002153 have a higher multiple of changes (|log_2_FC|≥ 4), indicating that these transcription factors actively responded to freezing defense responses.

**Table 1:** FPKM values of differentially expressed TF in different freezing times

Gene ID	Gene Family	B0	B3	B8	B24
unigene033097	NAC	217.4767	398.9967	622.0033	891.4433
unigene039744	CAMTA	0.7667	0.2833	0.4667	16.5967
unigene018905	bHLH	25.4633	14.5700	13.0667	5.3767
unigene009801	ZFP	1.1167	3.2733	4.2567	5.5167
unigene056021	WRKY	4.1567	13.5833	15.9000	18.1000

### Gene expression level validation by qRT-PCR

**Fig. 14:**
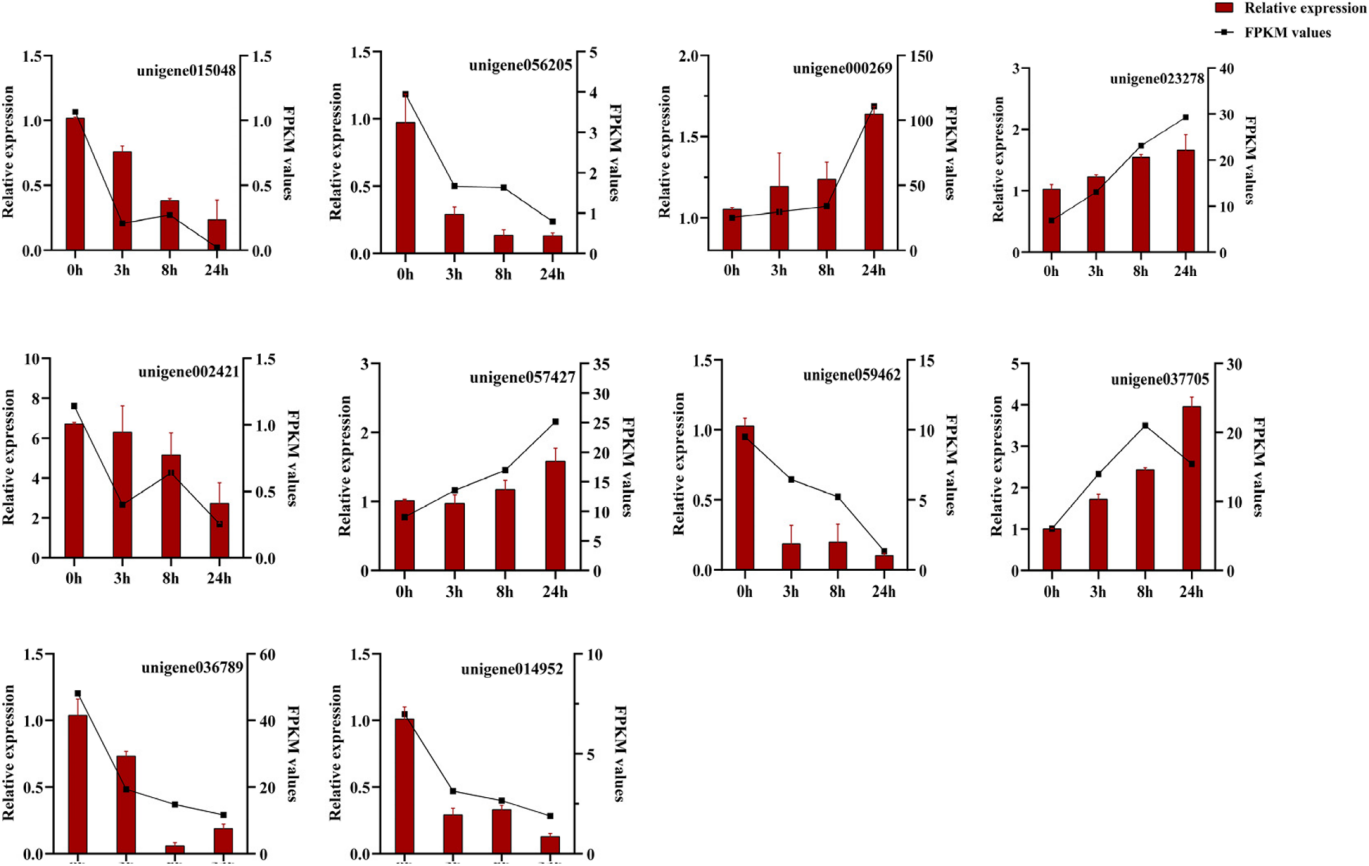
The qRT-PCR results of the DGEs related to freezing resistance. Error bars represent standard errors of the relative expression levels mean values by qRT-PCR (*n* = 3) (left y-axis). Broken lines represent the change in FPKM values of RNA-Seq (right y-axis)

To validate the quality of RNA-seq data, we selected 10 genes with a high fold-change in the “glucosyl/glucuronosyl transferases” family enriched in three stress stages for qRT-PCR analysis (Fig. 14). Results showed that the relative expression of unigene015048, unigene056205, unigene002421, unigene059462, unigene036789 and unigene014952 decreased significantly with the extended time. In addition, the expression levels of unigene000269, unigene023278, unigene057427 and unigene 037305 were increased significantly by freezing treatment. Although the fold-change in the expression level detected by qRT-PCR did not completely match the RNA-seq results, the expression patterns of all candidate genes under these two detection methods were similar.

## Discussion

### Changes to morphology, physiology and microstructure under freezing stress

When plants are subjected to low temperatures, the membrane should be firstly damaged [41]. The destruction of the membrane system may eventually lead to a large amount of solute outflow, matrix imbalance and metabolic disorder. At the same time, the lipid peroxidation produced abundant MDA, causing a cross-linking polymerization of proteins, nucleic acids and other macromolecules, which was toxic to cells [42, 43]. In addition, plants would suffer from different degrees of dehydration under low-temperature stress. Osmoregulation is an adaptive response to low-temperature stress by accumulating metabolites to raise the concentration of cellular fluids, reduce osmotic potential, and thus maintain normal cell turgor pressure and metabolism [44–46]. The soluble protein, soluble sugar and proline are important osmotic adjustment substances for plants to defense against freezing stress. Our results suggested that the response of MO seedlings to freezing stress was complex physiological changes. Over the 24 h course of freezing stress, there was an increase in REL, MDA and proline contents, and amount of soluble protein and sugar (Fig. 6). The CRT value decreased and the SR value increased, which suggests that the palisade tissue and spongy tissue of MO leaves exposed to freezing stress were damaged (Fig. 5). Compared with palisade cells, sponge tissue was more severely damaged (Fig. 4). Furthermore, the decrease of chlorophyll content may be related to inhibition of synthesis or acceleration of catabolism [47].

The antioxidant system reduces ROS and promote the freezing tolerance ability [48, 49]. Therefore, the increase of POD and SOD activities may play a critical role in eliminating ROS induced by low temperatures in MO leaves (Fig. 6). The roots of MO are abundant in oligosaccharides that are associated with the freezing resistance, such as *Panax ginseng* [50] and *Arnebia euchroma* [51] were also found this phenomenon. At the same time, the content of oligosaccharides in the roots of MO seedlings after freezing treatment was detected by HPLC-ELSD. However, the results showed that nystose was not detected, which may be accumulated gradually during the process of prolonged growth in MO. Taken together, these results of physiological changes indicated that MO seedlings had developed the ability to withstand short-period freezing stress, laying a foundation for its introduction to the north of Guangdong.

### Plant hormone signal transduction under freezing stress

Plant hormones not only participate in the process of growth and development, but also serve as a key endogenous factor mediating stress response. Plants resist and adapt to adversities by transmitting external environmental stimuli to multiple hormone signal transduction pathways [52, 53].

Studies show that freezing stress promoted the increase of ABA content, which further induced the expression of freezing response genes [54, 55]. PYR/PYL/RCAR receptors, located upstream of the ABA signal pathway, were important to recognize and start the primary process of ABA signal transduction [56]. After binding with ABA, PYR/PYL/RCAR receptors interacted with class A protein phosphatase PP2C, ABI and HAB to form PYR/PYL/RCAR-PP2C complexes, which inhibited the activity of protein phosphatase and started ABA signal transduction [57]. Our research showed that the expression level of *PYL4*(unigene036836) increased rapidly in the early stage of freezing stress in MO (Fig. 11). Ren et al. found that overexpression of *VaPYL4* gene could enhance the freezing tolerance in grape callus [58]. In addition, the freezing response depends on auxin homeostasis in different plant tissues. Polar auxin transport was regulated by AUX1/LAX inflow vectors, PIN efflux vectors and ABCB/MDR/PGP [59, 60]. We found that auxin response protein genes such as unigene030516, unigene044149, unigene014764 and unigene017859 were significantly induced by freezing stress (Fig. 11). Previous studies have shown that a considerable number of auxin regulation related genes were induced by low temperature [61–64]. Hence, auxin related DEGs may play an important role in MO responses to freezing stress.

### Transcription factor expression under freezing stress

Transcription factors (TFs) activate the expression of freezing responsive genes by binding to cis acting elements of the promoters, regulating signal transduction pathways in plants to improve freezing tolerance. The differential freezing response TFs of MO were significantly enriched in MYB, NAC and AP2/ERF families. AP2/ERF family members include AP2, RAV, ERF and DREB, among which DREB and ERF subfamily members are ideal candidate genes for crop improvement [65]. The pathways of plants response to freezing stress are divided into ABA-dependent and ABA-independent signal transduction pathways. There are two main types of DREB genes (*DREB1* and *DREB2*) in *Arabidopsis*. The *DREB1* genes are ABA-independent, and partial *DREB2* genes are ABA-dependent, while others are all involved in ABA-dependent response pathways [66, 67]. When plants were exposed to the freezing environment, they would firstly transmit low temperature signals in the form of Ca^2+^. The calmodulin binding protein activator (*CAMTA*) activated the expression of DREBs, which positively induced the expression of downstream freezing resistance genes dependent on DRE/CRT cis-acting elements [68, 69]. Our studies indicated that *CAMTA1* (unigene039744) gene was significantly induced in the MO freezing process (Table 1), which may further activate *DREB1* (unigene023745) gene to respond to low temperature signal transmission (Fig. 13a). In addition, we also found that *DREB2C* (unigene031863) gene was significantly up-regulated at the early stage and persistently overexpressed (Fig. 13a), suggesting that its mechanism of action might be related to ABA. Studies have shown that the *AmDREB2C* gene of *Ammopiptanthus mongolicus* responded strongly to low temperature. Overexpression of *AmDREB2C* enhanced the freezing tolerance in *Arabidopsis*, which was sensitive to exogenous ABA [70]. Lee et al. [71] found that *DREB2C* interacted with *ABF2*, which regulated the expression of ABA response genes. Overexpression of *ABF2* can enhance the freezing tolerance of transgenic *Arabidopsis* and affect ABA sensitivity. Therefore, the signal conduction mechanism of *DREB2C* may be related to ABA.

ERF family also plays an important role in response to low temperature. In this study, ERFs such as unigene039160, unigene029774, unigene016214 and unigene036025 were significantly induced by low temperature, among which *ERF1* (unigene039160) was continuously down regulated during the whole freezing process (Fig. 13a). Previous studies have illustrated that there was a complex interaction between ABA, ethylene and ERFs. For example, *ERF1*, *JERF1* and *TSRF1* regulated the biosynthesis of ABA, while ABA negatively regulated the expression of *ERF1* [72]. Besides, our studies suggested that *MYB4* (unigene048676) positively responded to freezing stress (Fig. 13b). *MYB4* reversely induced *PAL2*, *ScD9SAD* and *COR15a* promoters under low temperature, and its overexpression can enhance freezing resistance [73]. In addition, NAC genes were involved in different hormone signal transduction in the stress response process. Studies showed that the expression level of *OoNAC72* was significantly increased after exogenous ABA stimulation [74]. We found that the continuously up-regulated *NAC72* (unigene033097) may participate in the MO freezing response process through ABA dependent signal pathway (Table 1).

### Carbohydrate and proline metabolism under freezing stress

Carbohydrate metabolism is the basis for maintaining cell life. Carbon energy mainly comes from starch degradation. The enzymes and genes involved in starch degradation are crucial to carbohydrate metabolism. In this study, the “carbohydrate metabolism” pathway was significantly enriched in “starch and sucrose metabolism” and “pentose and glucuronate interconversions”. The expression of sucrose phosphate synthase (*SPS*, unigene029215) and fructose kinase (unigene027283) genes were significantly up-regulated after 24 h freezing stress (Fig. 12). Carbohydrate transport of advanced plants was mainly in the form of sucrose, which was synthesized by *SPS*. *SPS* catalyzes fructose 6-phosphate and UDPG to generate sucrose 6-phosphate, which generates sucrose through the catalysis of phosphorylase (*SPP*). *SPS* activity was positively correlated with sucrose accumulation and starch decomposition [75]. Thus, the soluble sugar content of MO increased under the continuous freezing treatment. Carbohydrate plays many roles in improving freezing resistance, including osmotic regulation, sugar signal transduction, and antioxidation [76].

Glycosylation is a modification reaction of natural compounds in plants. Glycosyltransferases (GTs) perform glycosylation, enabling substrate molecules to add sugar groups by catalyzing the formation of glycosidic bonds to form more stable natural glycosides or glycolipids [77]. A large number of studies have shown that GTs were involved in stress response, secondary metabolite synthesis and hormone regulation. For example, *UGT76F1* regulated the auxin level in *Arabidopsis* through IPyA glycosylation [78]; *CsUGT75C1* improved the cold resistance of tea plants by regulating the synthesis of anthocyanins [79]; *AhUGT83A1* was involved in the regulation of peanut resistance to drought, low temperature and salinity [80]. Interestingly, the “glucosyl/glucuronosyltransferase” family was significantly enriched after 24 h freezing treatment in this study. The expressions of unigene015048, unigene037705, unigene036789, unigene056205, unigene059462 and unigene014952 genes was significantly induced by low temperature (Fig. 14). The qRT-PCR results were consistent with the RNA-seq data. In addition, our previous research found that the “glucosyl/glucuronosyltransferase” family may participate in the biosynthesis of MO oligosaccharides, which speculated that the content of oligosaccharides increased, thus improving the freezing resistance of MO [81].

Proline accumulation is a self-regulation for plants to cope with low temperature stress. Both the decrease of proline degradation and the increase of proline synthesis are the main factors leading to its accumulation. When plants were exposed to stress, proteins would be largely decomposed into NH_3_, and excess NH_3_ was toxic to cells. Plants converted NH_3_ into glutamate and ornithine through various amino acid exchanges, amino acylation and transaminases, and finally synthesize proline [82]. Δ^1^-Dihydropyrrol-5-carboxylate synthase (*P5CS*) and proline dehydro-genase (*ProDH*) are crucial for proline synthesis and degradation [83]. In this study, the expression of the *ProDH* (unigene009207) gene was significantly downregulated at the early stages of freezing stress (3 ~ 8 h), indicating that low temperature inhibited its expression and alleviated the degradation of proline at the transcriptional level (Table S8). However, the expression of the *P5CDH* gene was no significant change, suggesting no synergistic effect between *P5CDH* and *ProDH* in improving MO freezing resistance. At the same time, the expression of glutamine synthetase (*GS*, unigene059066) was also significantly down-regulated, hindering the synthesis of glutamine and inhibiting the process of ammonium assimilation. Therefore, we inferred that the proline content of MO increased by inhibiting the degradation process, rather than by promoting its synthesis process. The proline content in MO increased sharply at the early freezing stage, and then its content decreased, but remained higher than 0 h, which was consistent with the above results. Meanwhile, the increase of proline would negatively regulate the expression of the *P5CS* (unigene024763) gene [84].

## Conclusions

In this study, physiological and transcriptomic analyses were performed to reveal the potential mechanisms by which MO responded to freezing stress. The results showed that leaf dehydration, palisade cell and spongy mesophyll destruction occurred when MO seedlings were exposed to -2 °C. The increased REL and MDA content suggested that the membrane of MO was damaged. A decrease in chlorophyll content indicated that the chloroplasts were damaged. Meanwhile, low temperature stimulated the freezing resistance response of MO, and the content of a series of osmoregulatory substances such as proline, soluble sugar and soluble protein were increased, as well as the activity of antioxidant enzymes such as SOD and POD were up-regulated. The dynamic change pattern of oligosaccharides in roots of MO under freezing stress would require further study. Furthermore, the molecular freezing response mechanism of MO may be related to plant hormone signaling transduction, transcription factor regulation, and gene expression of carbohydrate and proline metabolic pathways. Our research provides new insight into antifreeze molecular breeding of MO and lays a foundation for improving the quality of MO.

### Supplementary Information

**Additional file 1: Table S1.** Primer sequences used for qRT-PCR. **Table S2.** Difference of microstructure parameters of MO leaves under different freezing time. **Table S3.** The physiological changes of MO seedlings under freezing stress. **Table S4.** Quality control of transcriptome. **Table S5.** FPKM values of DEGs related to plant hormone signal transduction pathway. **Table S6.** FPKM values of DEGs related to starch and sucrose metabolism pathway. **Table S7.** FPKM values of DEGs related to AP2/ERFs and MYBs. **Table S8.** FPKM values of DEGs related to proline synthesis and degradation pathways. **Table S9.** The results of qRT-PCR.

**Additional file 2: Fig. S1. **The distribution of unigenes sequence length. **Fig. S2. **Number of gene annotations among different databases. **Fig. S3. **Gene quantity distribution of each species.

## Data Availability

RNA sequence data were deposited in NCBI under the BioProject accession number PRJNA944555.
