# Supplementary material for: Physiological and transcriptomic analysis reveals the potential mechanism of Morinda officinalis How in response to freezing stress
Source: BMC Plant Biol. 2023 Oct 23;23:507. doi: 10.1186/s12870-023-04511-5 (PMC10591367; doi:10.1186/s12870-023-04511-5)
Supplement: Supplementary file 2 — Additional file 2: Fig. S1. The distribution of unigenes sequence length. Fig. S2. Number of gene annotations among different databases. Fig. S3. Gene quantity distribution of each species. [file 12870_2023_4511_MOESM2_ESM.docx]

Physiological and Transcriptomic Analysis Reveals the Potential Mechanism of *Morinda officinalis* How in Response to Freezing Stress

Zhenhua Luo, Xiaoying Che, Panpan Han, Zien Chen, Zeyu Chen, Jinfang Chen, Sishi Xiang, Ping Ding^*^

School of Pharmaceutical Sciences, Guangzhou University of Chinese Medicine, Guangzhou 510006, China

* Correspondence: Ping Ding

[dingping@gzucm.edu.cn](mailto:dingping@gzucm.edu.cn)


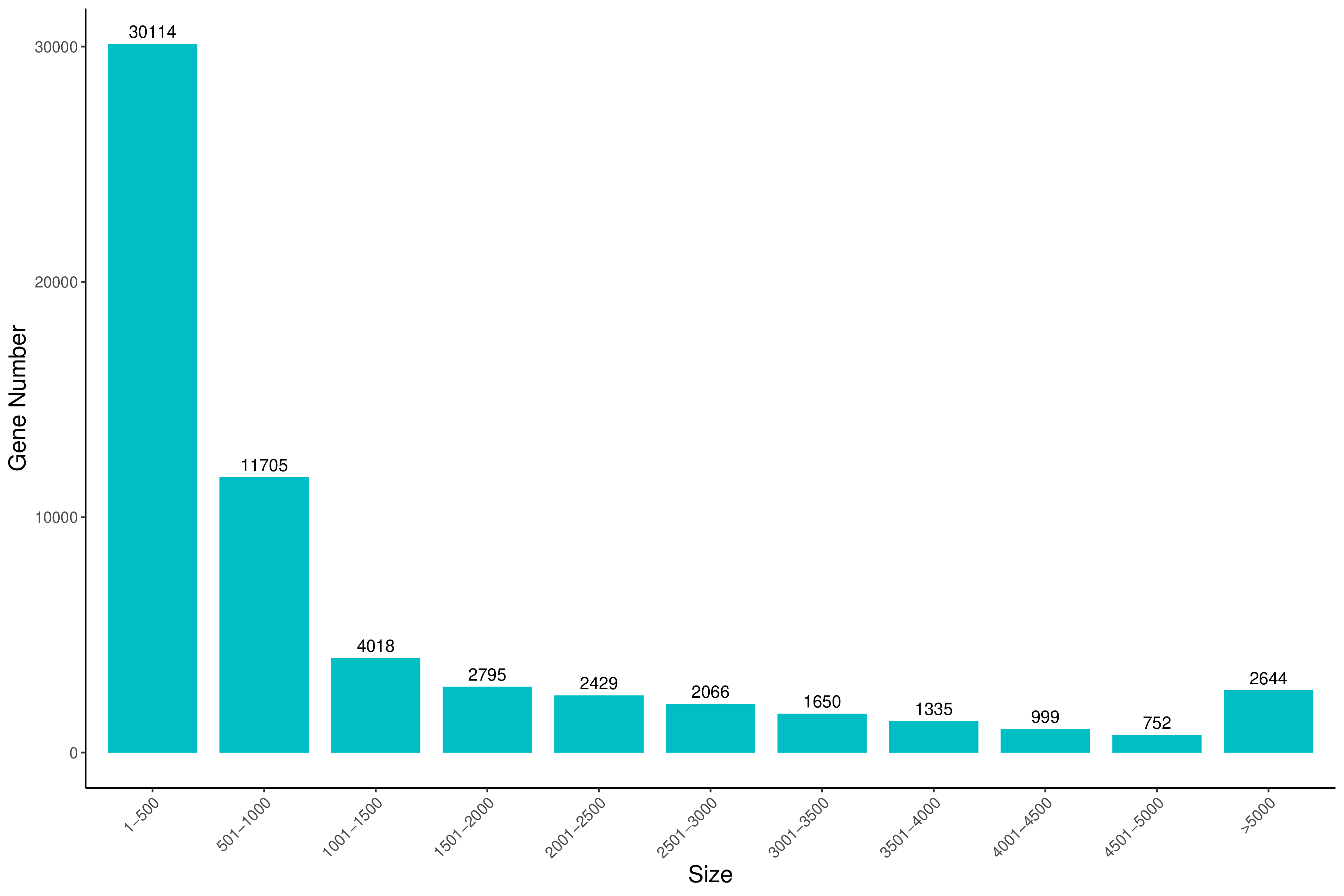


**Fig. S1** The distribution of unigenes sequence length.


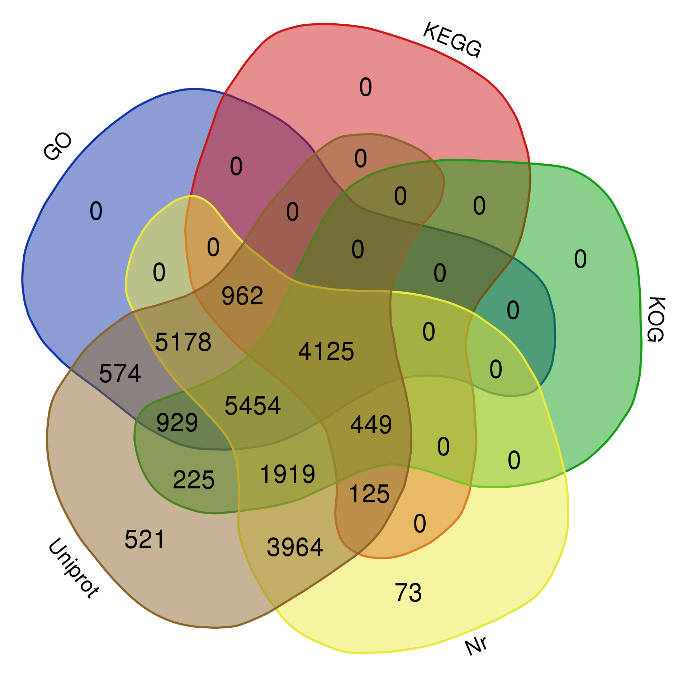


**Fig. S2** Number of gene annotations among different databases.


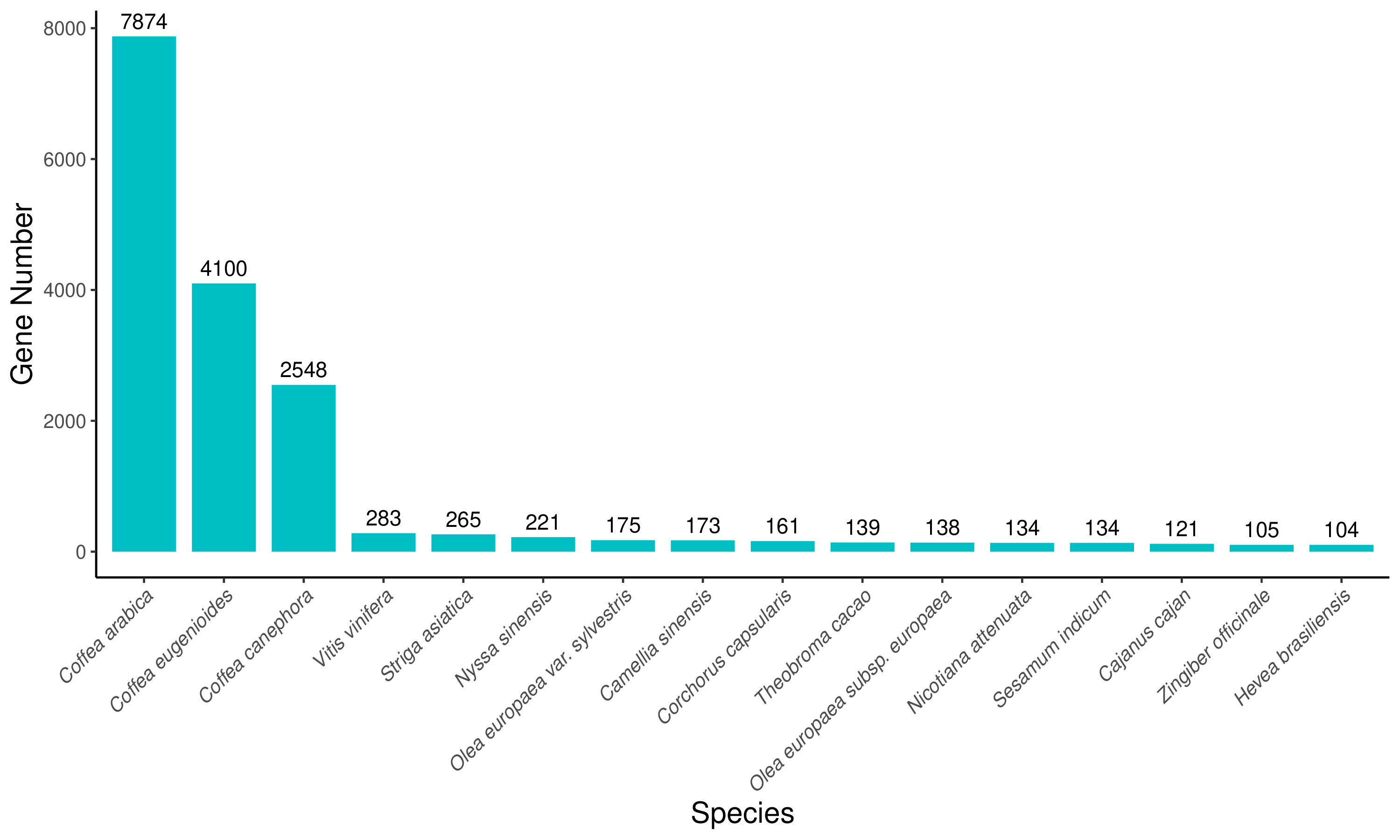


**Fig. S3** Gene quantity distribution of each species.
